# Coupling of Surface Plasmon Modes and Refractive Index Sensitivity of Hollow Silver Nanoprism

**DOI:** 10.1038/s41598-018-34477-6

**Published:** 2018-10-30

**Authors:** K. J. Zhang, D. B. Lu, B. Da, Z. J. Ding

**Affiliations:** 1 Key Laboratory of Strongly-Coupled Quantum Matter Physics, Chinese Academy of Sciences; Hefei National Laboratory for Physical Sciences at Microscale and Department of Physics, University of Science and Technology of China, Hefei, Anhui 230026 P. R. China; 2 Center for Materials Research by Information Integration, Research and Services Division of Materials Data and Integrated System, National Institute for Materials Science, 1-2-1 Sengen, Tsukuba Ibaraki, 305-0047 Japan

## Abstract

Localized surface plasmon (LSP) modes depend strongly on the morphology of nanoparticle and the surrounding dielectric medium. The hollow nanostructure provides a new way to modulate the surface plasmon modes due to the additional cavity surface. In this work, we study systematically the multipolar surface plasmon modes of hollow silver nanoprism (HSN) by simulation of electron energy loss spectroscopy (EELS) spectra based on the boundary element method (BEM). Herein the effects of the cavity size and position are taken into account. The LSP modes of HSNs are compared with those of perfect silver nanoprism (SN). The red-shift behaviors of multipolar modes can be found as increasing the cavity size. Modes *A* and *C* have similar red-shift tendency and obey the plasmon ruler equation, which can be explained by dipole-dipole coupling mode. Meanwhile, the degenerate modes will be split by changing the cavity position, and opposite shift tendencies of split degenerate states are observed. These are caused by different coupling nature of degenerate modes. Moreover, high refractive index sensitivity (RIS) can be obtained for HSN by changing the cavity size and position.

## Introduction

The optical properties of noble metals at the nanoscale have gained considerable attention during the last decade^1–6^. These benefit from the excitations of localized surface plasmon (LSP) modes, which allow the electromagnetic energy to be enhanced around nanoparticle surface as collective oscillations of conduction band electrons at surface. The locally enhanced electric field of LSP has been widely applied to surface enhanced Raman spectroscope (SERS)^7^, which can extremely enhance the Raman signal of measured molecules and detect the molecules more easily. Meanwhile, the resonance frequency of LSP mode is highly sensitive to the geometric parameters of nanoparticles (i.e. size and shape) and the surrounding dielectric environment^8^. These features make it possible for metal nanoparticles to be used in the sensor^9^, nano-photonics^10^ and solar cells^11^. Recently, the strong interaction between LSP and microcavity reveals the promising applications in quantum emitter and quantum plasmonics^12,13^.

To study LSP modes, electron energy loss spectroscopy (EELS) technique is an extremely advantageous method owing to its outstanding spatial (<1 nm) and energy resolution (<100 meV)^14–16^. In the frame of scanning transmission electron microscopy combined with electron energy loss spectroscopy (STEM-EELS), the LSP modes of nanoparticles are excited by high energy incident electrons and are related to the position of incident electron beam (especially for nanoparticle with lower symmetry). Besides, for nanoparticle dimer, the out-of-phase (antibonding) coupling of dipole mode ($\to \leftarrow$) can also be excited in STEM-EELS, but not for plane wave excitation^17,18^. On the other hand, EELS map is supposed to be able to map the electronic local density of LSP modes^19,20^. Two-dimensional (2D) EELS map has been shown to reflect the local density of states (LDOS) along the *z*-axis under a quasistatic approximation^21^. Generally, EELS maps can be used to characterize LSP modes. Recently, Nicoletti *et al*. have developed three-dimensional (3D) imaging technique to visualize LSP modes by STEM-EELS^22^. Hohenster *et al*. have also introduced a tomography scheme based on the EELS to reconstruct the 3D LDOS of LSP mode beyond the quasistatic approximation^23–25^.

Previously, most of the works concentrate on the LSP modes of single nanoparticle with different shapes (e.g. sphere^26^, cube^27,28^, rod^29^ and prism^30–32^) and dimer configuration (two adjacent nanoparticles)^33–35^. The coupling interaction between the adjacent nanoparticles can give rise to locally enhanced electric field (i.e. hot spot). For dipole-dipole coupling case, the strength of coupling depends on the spacing distance of dimer, and the relative plasmon shift decays exponentially with distance (so-called plasmon ruler)^34^. Actually, changing the spacing distance is not easy for practical application. In recent years, the hollow nanostructures have received much attention thanks to additional surface of the cavity. Arbiol^36^ *et al*. showed that the LSP resonance of hollow cuboid metal nanostructures can be manipulated from ultraviolet to near-infrared. Similarly, Yazdi^37^ *et al*. reported plasmon tuning in hollow AgAu nanorods. The coupling between the inner surface and the outside surface results in tunable optical properties. In addition, the existed cavity may break the symmetry of the original nanoparticle. Thus, the degenerate modes may be split. Among the various hollow nanostructures, hollow triangular nanoprism is attractive: the high-order modes of perfect triangular nanoprism can be excited easily due to lower symmetry *D*_3v_ (compared with sphere and cube); meanwhile, the nanoprisms possess larger dielectric sensitivity factor compared to nanosphere and nanocube^30,38,39^. The refractive index sensitivity (RIS) is defined as the shift in the wavelength (Δ*λ*) of LSP mode per unit in the refractive index change of the surrounding medium (RIS = Δ*λ*/Δ*n*). The high RIS value of LSP mode is desired for detection of dielectric environment changes, and forms the basis of localized surface plasmon resonance spectroscopy^40^. Previous studies show that the RIS depends on the shape and size of nanoparticle^30,41^. Anisotropic nanoparticles and hollow nanoparticles can exhibit the larger RIS^42,43^. Since the nanoparticles with large RIS are very sensitive to the surrounding environment, they are expected for sensing biological molecules such proteins and antibodies. Similar sensing mechanism is also found in single nanoparticle detection by using optical microcavities, due to strongly enhanced light-matter interaction^44^.

The effects of the cavity (i.e. size and position) of hollow nanoprism on multipolar LSP modes are less well studied. In this work, we focus on the multipolar LSP modes of the hollow silver nanoprism (HSN) by using boundary element method. Here, the shape of cavity is chosen as circle, which is very common in experimental synthesis. The effect of the cavity size on multipolar LSP modes is studied by changing the cavity size gradually. Comparisons of LSP modes are made between a perfect silver nanoprism (SN) and a HSN. Analyzes of LSP modes are performed with the help of EELS maps and eigenmodes. The shifts of coupling modes will be explained based on hybridization model. Furthermore, the influence of the cavity position on multipolar LSP modes is also discussed. We have found that the degenerate modes can be split by varying the cavity positions. In addition, the refractive index sensitivity (RIS) of HSN for each LSP mode is also studied. Simultaneously, the effects of both of cavity size and position on RIS are taken into account. These results introduce possibilities for applications in sensors and functional plasmonic devices.

**Figure 1:**
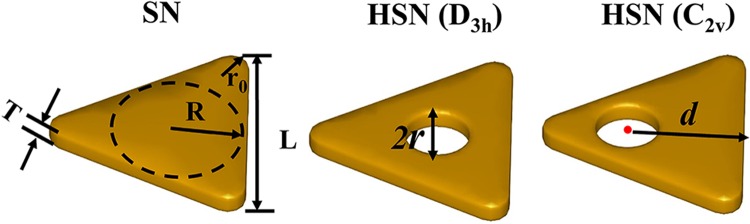
Schematics of three triangular nanoprisms (from left to right): (i) perfect silver triangular nanoprism (SN), (ii) hollow silver nanoprism with center-located cavity (*D*_3h_ symmetry), (iii) hollow silver nanoprism with a cavity located on median line (*C*_2v_ symmetry).

In this work, we focus on the hollow silver nanoprism. The schematics of triangular nanoprisms are shown in Fig. 1. Figure 1(i) shows the perfect triangular SN. The edge length (*L*) and thickness (*T*) of the triangular nanoprism are fixed at 55 nm and 5 nm, respectively. The radius of inscribed circle is *R*. Herein, the corners of triangular nanoprism are rounded, and the circular arc radius of the corner is r_0_ (we set *r*_0_ = 3 nm). Figure 1(ii) shows hollow SN, and the cavity (diameter 2*r*, 0 ≤ *r* < *R*) is locate in the center of the regular triangle. In this case, its symmetry is *D*_3h_, which is the same as SN. Varied positions of hole correspond to HSN with *C*_2v_ symmetry, as shown in Fig. 1(iii). Here *d* is the distance between the center of hole and the bottom edge. In this paper, the effects of holes (size and position) on LSP modes are studied. Besides, the effect of surrounding medium on LSP modes of HSNs is also discussed. And the effects of substrate and rounded corner on RIS will be taken into consideration. The energy of incident electron is set as 100 keV. The dielectric constants of Ag used in our simulations are taken from Johnson and Christy’s data^45^.

## Results and Discussion

### Influence of cavity size

**Figure 2:**
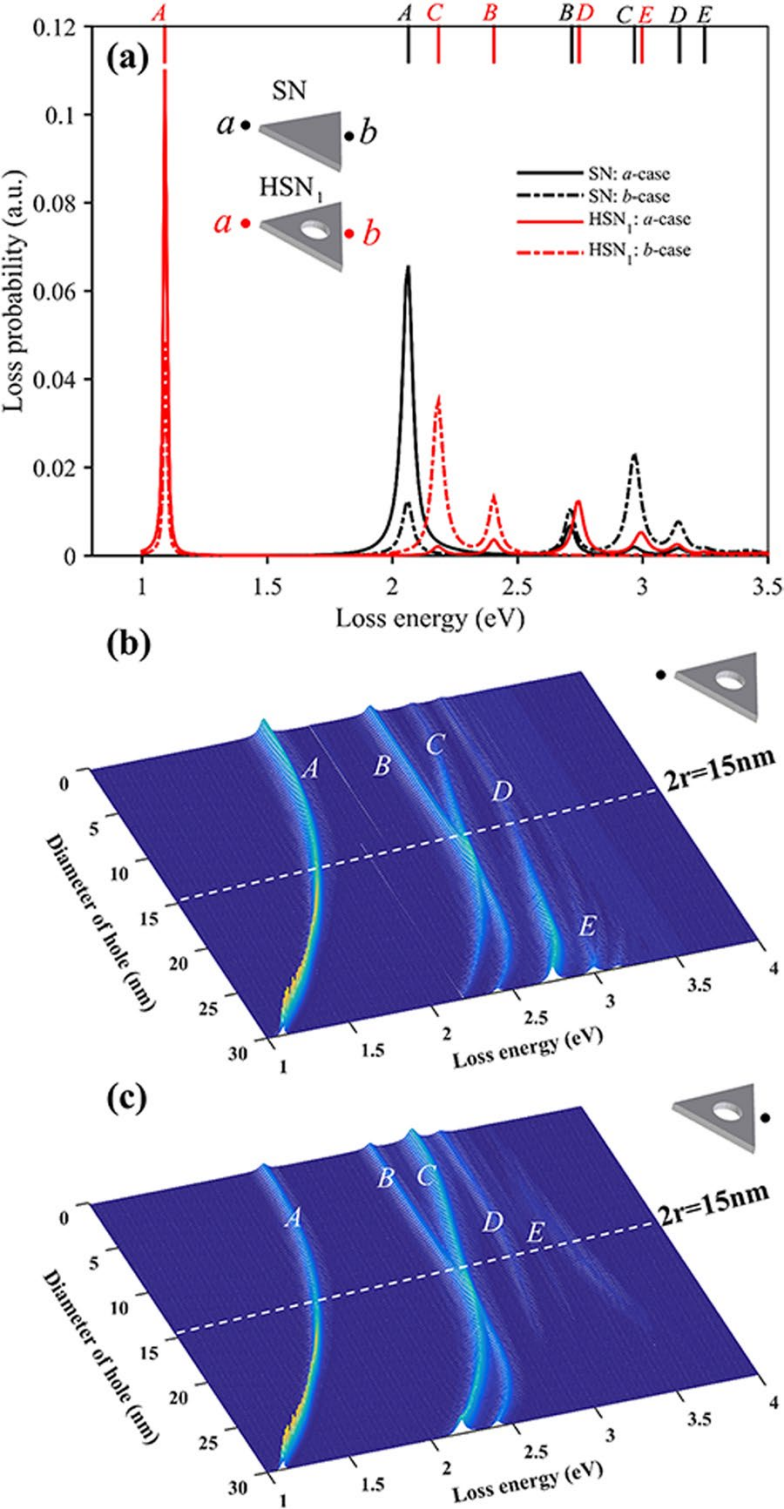
(**a**) Simulated EELS spectra taken at two electron beams for SN and HSN_1_ (2*r* = 30 nm). Each mode is labeled on the top of the figure: for SN case (black lines), mode *A* (2.064 eV), *B* (2.716 eV), *C* (2.967 eV), *D* (3.147 eV), *E* (3.247 eV); for HSN_1_ case (red lines), mode *A* (1.091 eV), *C* (2.184 eV), *B* (2.405 eV), *D* (2.746 eV), *E* (2.997 eV). (**b,c**) Simulated EELS spectra of HSNs by varying cavity size for electron beam incidence in *a* and *b* cases, respectively.

**Figure 3:**
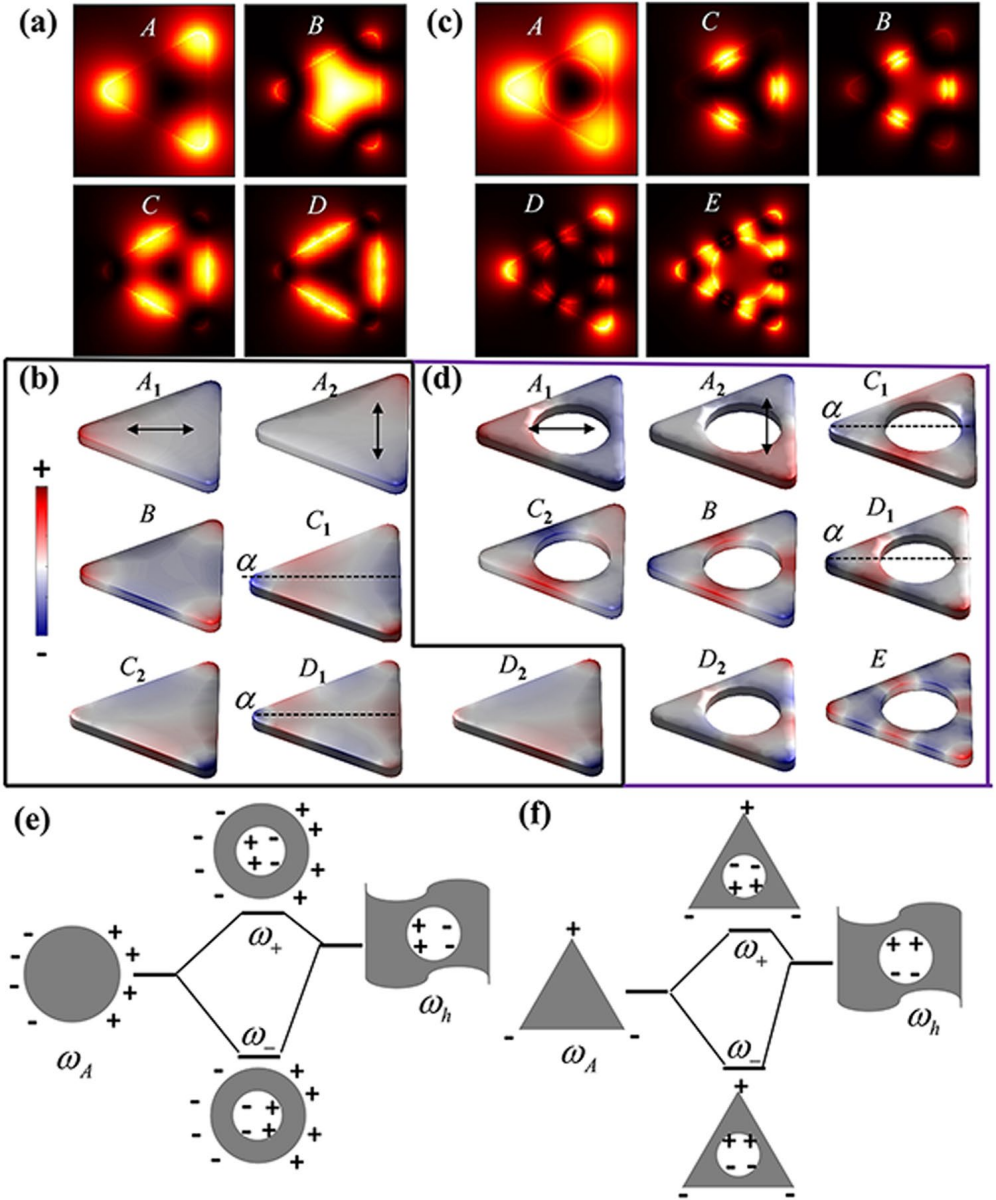
(**a**) Simulated EELS maps of modes *A*-*D* for SN. (**b**) Eigenmodes of the four modes for SN, some of them are degenerate: mode *A*_1_ and *A*_2_, mode *C*_1_ and *C*_2_, mode *D*_1_ and *D*_2_. These degenerate states are axisymmetric and antisymmetric referring to *α*-axis (dashed line). (**c**) EELS maps of modes *A*-*E* for HSN_1_. (**d**) Eigenmodes of modes *A*-*E* for HSN_1_, modes *A*, *C*, *D* all have degeneracy in the same way. (**e, f**) Schematic energy-level diagram, showing the plasmon coupling between outer surface (circle or triangle) and inner surface. Two coupling modes correspond to in-phase mode *ω*_−_ (**e**) and out-of-phase mode *ω*_+_ (**f**).

Firstly, we investigate the effect of cavity size on LSP modes of HSNs (*D*_3h_). Figure 2(a) shows the simulated EELS spectra of two nanoprisms, SN and HSN_1_ (diameter of hole *2r* = 30 nm). As we know, the LSP modes excited by electrons depend on the position of electron beam. Here, we have performed the calculations for two different incident electron beam positions (*a*-case and *b*-case). The direction of incident electron beam is perpendicular to the plate and the dots represent the coordinates of incident electrons on *xy*-plane. In EELS spectra of SN case, four distinct peaks can be found and are labeled as mode *A* (2.05 eV), *B* (2.71 eV), *C* (2.98 eV) and *D* (3.15 eV), respectively. Another weak shoulder can also be found from EELS signal of *b*-case, and is labeled as mode *E* (3.25 eV). Spatial intensity distribution of EELS is widely adopted to characterize the surface plasmon mode. The simulated EELS maps of the first four modes are shown in Fig. 3(a). Previous studies show that EELS map reflects the intensity of electric field distribution along the *z*-axis induced by LSP modes^19,46^. Thus, EELS map also shows the contact with surface charge distribution of LSP modes. For SN case, our simulation results are similar to those in ref.^47^. Experimentally, modes *A* and *C* of triangular nanoplate can also be measured by STEM-EELS technique^48–50^. As for mode *B*, it’s not easy to be identified due to the wide spread and weak signal of experimental EELS spectra. Keast^51^ *et al*. reported that higher order LSP mode (mode *D*) can be excited via experimental EELS. To further understand LSP modes, the intrinsic surface charges of LSP modes are obtained by solving the eigenmode equation, Eq. (3). Figure 3(b) shows the corresponding intrinsic surface charges of the modes *A*-*D*. With the help of eigenmode analysis, we find that modes *A*, *C* and *D* are all degenerate. For dipolar mode *A*, the surface charge distribution gathers at the vertices. There are two degenerate modes: mode *A*_1_ (dipole orientation →) and *A*_2_ (dipole orientation ↑). However, these two degenerate modes cannot be identified by EELS map due to *D*_3h_ symmetry. For charge distribution of mode *B* with *D*_3h_ symmetry, same charges concentrate on the three corners and opposite charges on the edges. The net charge polar moment is zero. Therefore, mode *B* is invisible in light-driven case. Two degenerate states of mode *C* are axisymmetric mode *C*_1_ and antisymmetric mode *C*_2_ referring to *α*-axis (see the dashed line in Fig. 3(b)). Similarly, for mode *D*, there are also two degenerate states: axisymmetric mode *D*_1_ and antisymmetric mode *D*_2_. Besides, EELS spectra of HSN_1_ (diameter 2*r* = 30 nm) show that all modes are red-shifted as compared the SN, as shown in Fig. 2(a). The corresponding EELS maps and intrinsic charge distributions are shown in Fig. 3(c) and (d), respectively. Compared to the SN case, EELS map of each mode for HSN_1_ is changed since the charge distributions change by coupling effect.

**Figure 4:**
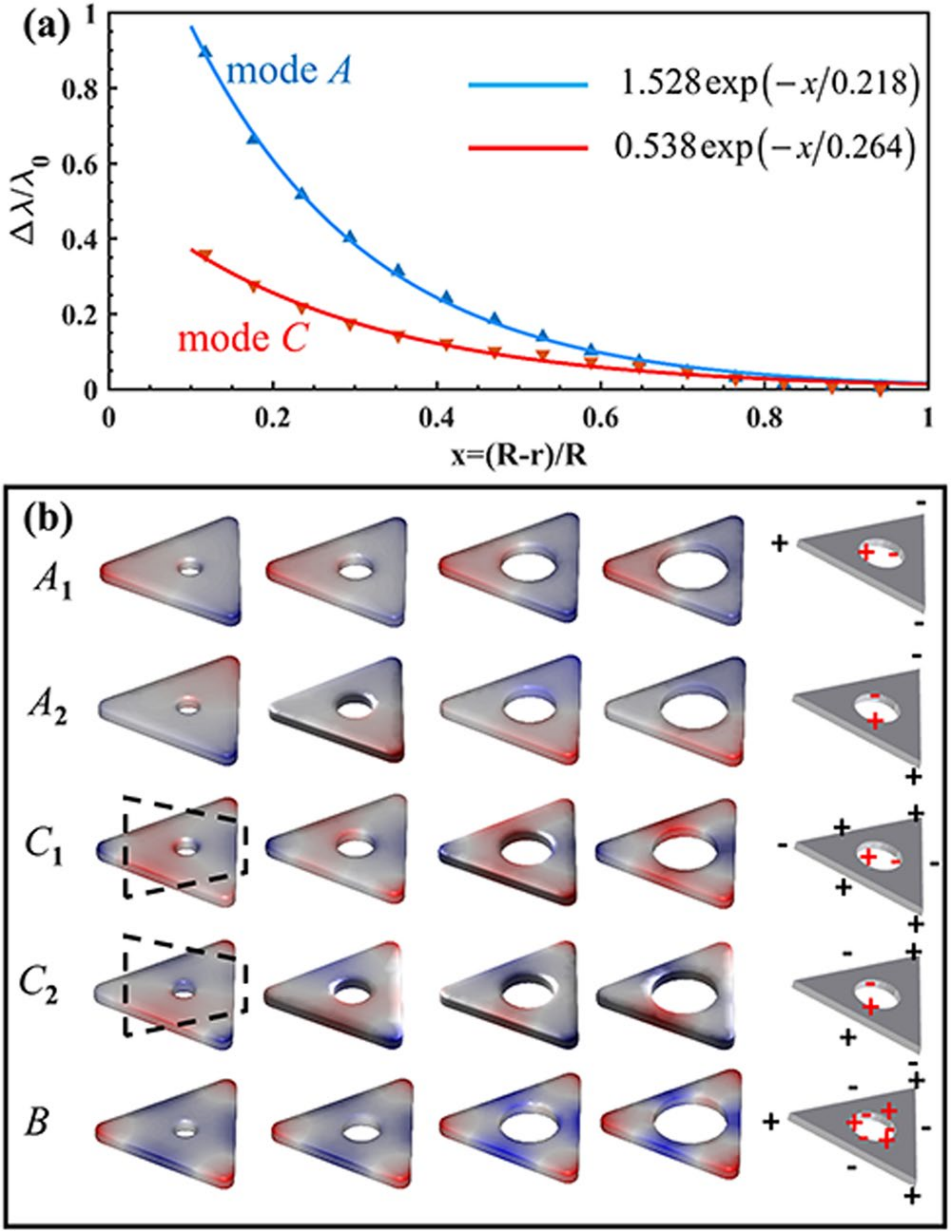
(**a**) Relative wavelength shift vs (*R* − *r*)/*R* for modes *A* and *C*; the fitted exponential relationships (*y* = *a* exp (−*x*/*τ*)) are shown as blue and red lines, respectively. (**b**) The eigenmodes (*A*_1_, *A*_2_, *C*_1_, *C*_2_, *B*) of HSNs with different cavity diameters (from left to right: 8, 14, 20, 25 nm). The schematic on rightmost column shows charge distribution of each mode (different colors are used for outer and inner surface).

To further clarify the cavity size effect, Fig. 2(b) and (c) show the simulated EELS spectra of HSNs varying with hole diameter (from 0 nm to 30 nm) for two different electron beam incidences, i.e. *a-* and *b*-cases, respectively. It is intuitive to trace the evolution of each mode with the change of cavity size. With increasing diameter of hole, a strong red-shift is observed for modes *A* and *C*. It is worth mentioning that the degenerate states are not split when increasing diameter. This feature can be explained based on a hybridization model for the plasmon modes. Pordan *et al*.^52^ presented the hybridization model to understand the LSP modes of nanoshell. They pointed out that LSP modes of nanoshell arise from the interaction between the sphere and hole plasmons. One coupling mode is symmetrically in-phase *ω*_−_ mode, and the other is out-of-phase *ω*_+_ mode, as shown in Fig. 3(e). This model can also be used to understand LSP modes of HSN. Similarly, Fig. 3(f) shows the schematic describing the hybridization of dipolar modes between the outer surface of triangular nanoprism and spherical cavity surface. The energy of in-phase *ω*_−_ mode will decrease as compared to primitive modes. Actually, mode *A* of HSN is the in-phase *ω*_−_ mode. For out-of-phase *ω*_+_ mode with higher energy, its signal intensity in EELS spectra is quite weak, and the energy of *ω*_+_ mode will blue shift with increasing the cavity size (see the weak blue-shifted signal in Fig. 2(c)). This blue-shifted feature is similar to out-of-phase coupling mode for dimer case ($\to \leftarrow$ mode)^53^. As we know, the plasmon ruler equation is widely used to describe coupling mode shift vs spacing distance of nanoparticle dimer^34^. It can be expressed as:

1 $$\frac{{\rm{\Delta }}\lambda }{{\lambda }_{{\rm{0}}}}=a\,\exp (-\frac{s/L}{\tau }),$$

where Δ*λ*/*λ*_0_ is relative plasmon shift, *s* is the spacing distance, *L* is the characteristic length of nanoparticle (i.e. diameter for sphere, edge length for cube), *a* and *τ* determine respectively the magnitude of plasmon shift and the decay of coupling mode with separation. Here, in HSN case, we found that modes *A* and *C* obey the plasmon ruler ${\rm{\Delta }}\lambda /{\lambda }_{0}=a{e}^{-x/\tau }$ well. Different from nanoparticle dimer case, we chose the relative distance *x* as (*R* − *r*)/*R*, which is the closest intersurface distance. The fitting parameters are shown in Fig. 4(a). Hazra^54^ *et al*. reported the similar feature of mode *A* for Au nanoprism with cavity. The degenerate eigenmodes of modes *A* and *C* for HSNs with different hole sizes are shown in Fig. 4(b). It is obvious that modes *A*_1_ and *A*_2_ belong to dipole-dipole coupling (transverse in-phase .. and longitudinal in-phase $\uparrow \uparrow$). For modes *C*_1_ and *C*_2_, it is interesting that charges located on the outside vertexes do not participate in the coupling (the charges on vertexes almost unchanged, see *C*_1_ and *C*_2_ in Fig. 4(b)). The coupling effect seems to arise primarily from interaction between outside edges and inner cavity surface (see dashed box area in Fig. 4(b)). Thus, this is equivalent to dipole-dipole interaction ($\to \to$ for *C*_1_, $\uparrow \uparrow$ for *C*_2_). That is why mode *C* has dipole-like exponential decay relationship, and decay constant *τ*_*C*_ closes to *τ*_*A*_.

In addition, for mode *B*, the energy is almost unchanged for the diameter 2*r* < 20 nm. The analysis of charge distributions shows almost no coupling between outer and inner surface when the diameter less than 20 nm (see mode *B* in Fig. 4(b), three ones on the left). The charge distribution pattern is similar to the primitive mode *B* (shown in Fig. 3(b)). When the diameter is greater than 20 nm, inner surface closes to the outer edges, and charges on inner surface start to interact with charges located on both vertexes and edges of outer surface. That is the coupling between hexapolar mode of cavity surface and primitive mode *B* of triangular surface (see mode *B* in Fig. 4(b), two ones on the right). There are also slight red-shift for modes *D* and *E*. This is owing to coupling of high-order modes: quadrupole-quadrupole interaction for mode *D* and hexapolar-hexapolar interaction for mode *E*. As shown in Fig. 3(d), for modes *D* and *E*, the charges located on inner and outer surfaces show the characteristic of multipole distribution. The quadrupole (hexapolar) interaction energy is weaker than the dipole interaction. Thus, the redshift of high-order modes will be much weaker than dipole mode.

### Influence of cavity position

**Figure 5:**
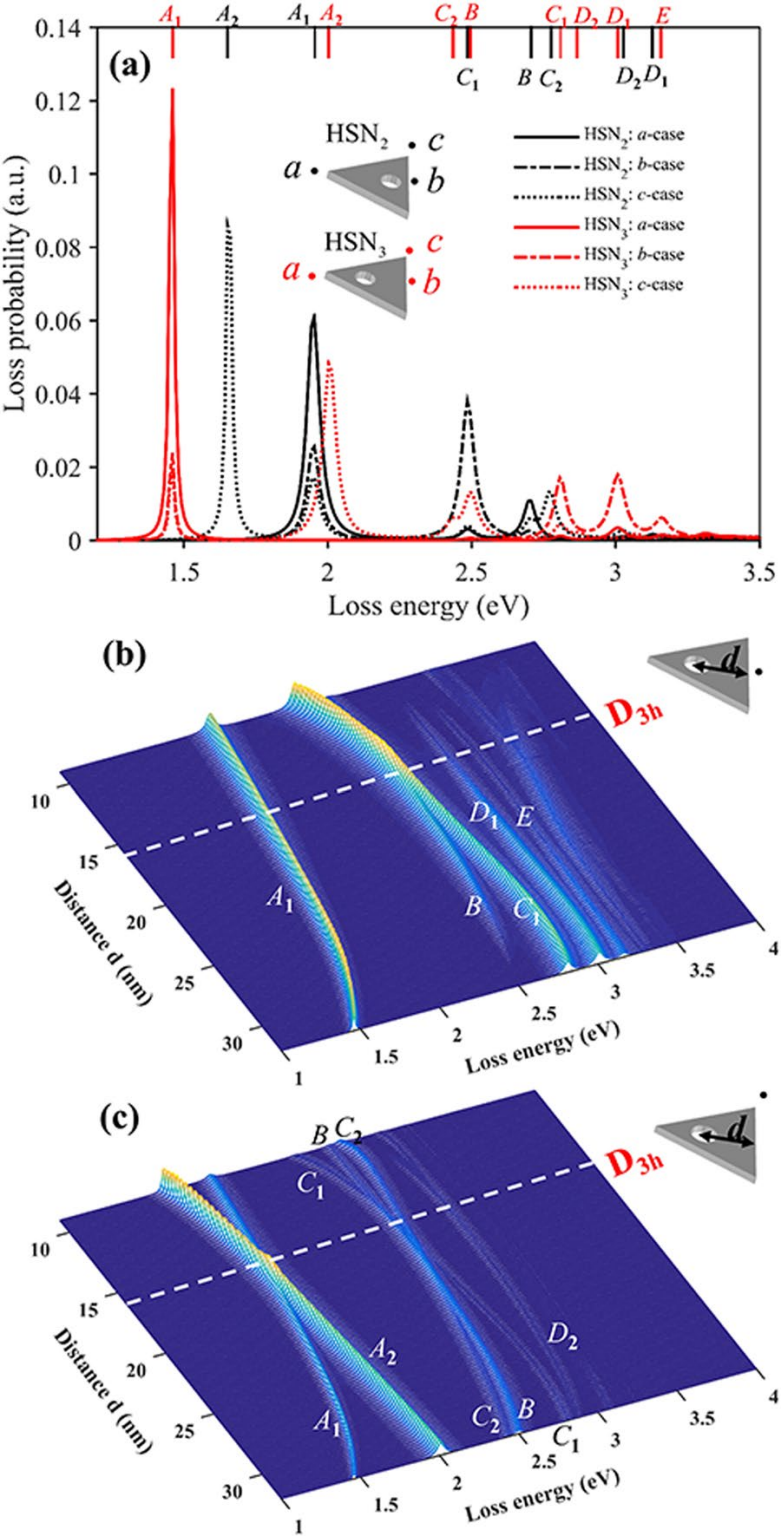
(**a**) Simulated EELS spectra taken at three electron beams for HSN_2_ (*d* = 9 nm) and HSN_3_ (*d* = 32 nm) with cavity diameter 2*r* = 15 nm. Each mode is labeled on the top of the figure: for HSN_2_ case (black lines), mode *A*_2_ (1.652 eV), *A*_1_ (1.953 eV), *C*_1_ (2.485 eV), *B* (2.706 eV), *C*_2_ (2.776 eV), *D*_2_ (3.027 eV); for HSN_3_ case (red lines), mode *A*_1_ (1.462 eV), *A*_2_ (2.003 eV), *C*_2_ (2.435 eV), *B* (2.495 eV), *C*_1_ (2.806 eV), *D*_2_ (2.866 eV), *D*_1_ (3.007 eV), *E* (3.157 eV). (**b,c**) Simulated EELS spectra of HSNs by varying cavity position for electron beam *b* and *c*, respectively.

**Figure 6:**
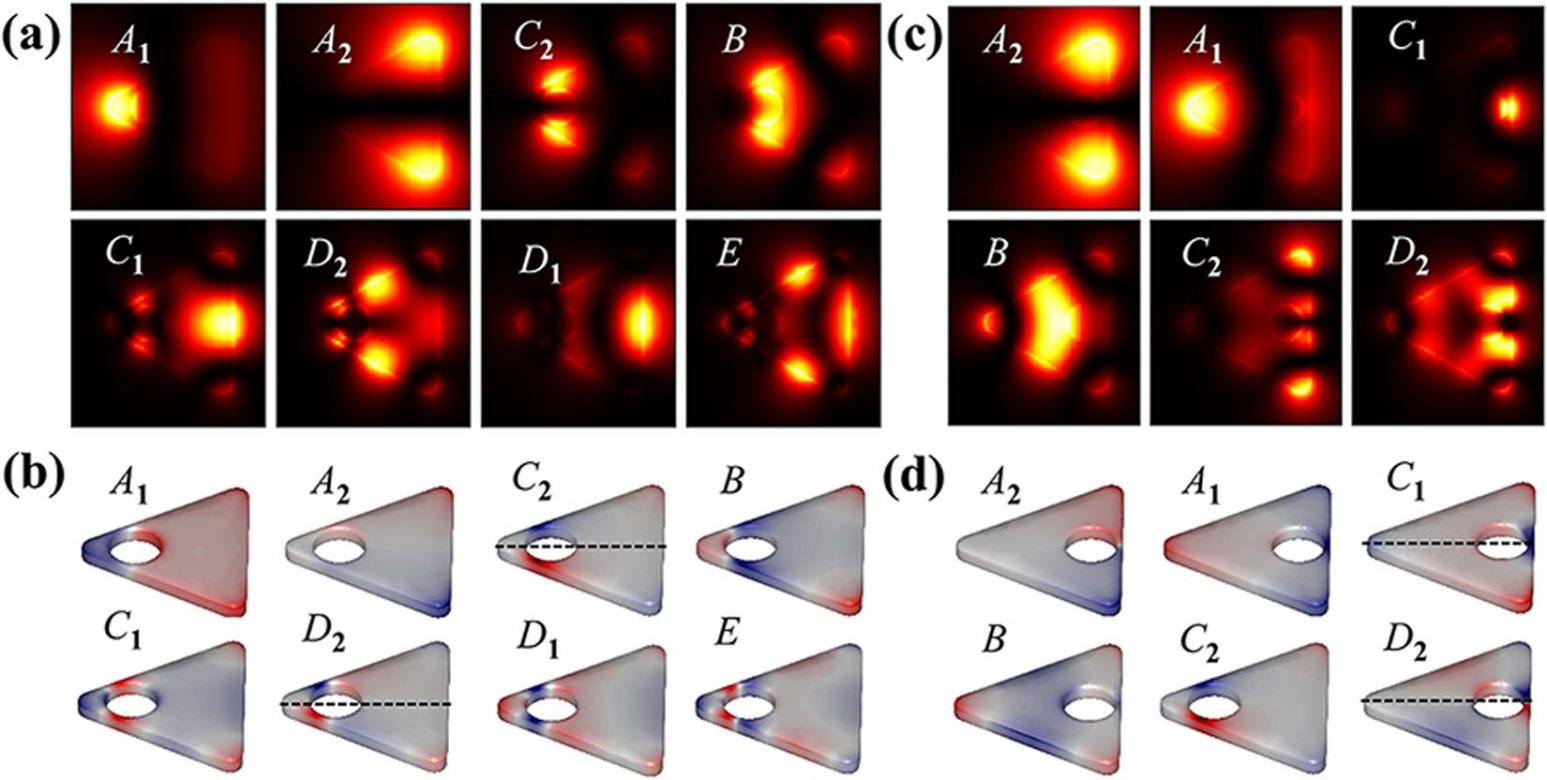
(**a,b**) Simulated EELS maps and eigenmodes of modes *A*_1_, *A*_2_, *C*_2_, *B*, *C*_1_, *D*_2_, *D*_1_ and *E* for HSN_3_ respectively; (**c,d**) Simulated EELS maps and eigenmodes of modes *A*_2_, *A*_1_, *C*_1_, *B*, *C*_2_ and *D*_2_ for HSN_2_ respectively.

In this section, we investigate the effect of cavity position on LSP modes. The center of cavity is varied along the median line of triangle, and the distance between the center of cavity and the bottom edge is marked as *d* (see HSN (*C*_2v_) in Fig. 1). Here we considered the HSN with fixed cavity diameter of 2*r* = 15 nm. For HSN with *D*_3h_ symmetry, we noticed that the resonance energies of modes *B* and *C* become congruent when 2*r* = 15 nm (see white line in Fig. 2(b,c)). Thus, we are wondering that what will happen if centers of the triangle and the cavity are misaligned. In this case, the HSN will has lower symmetry (*C*_2v_ symmetry). Figure 5 (a) shows the simulated EELS spectra of HSN_2_ (*d* = 9 nm) and HSN_3_ (*d* = 32 nm), performed at three different electron incident positions (*a*, *b* and *c* cases). EELS maps and eigenmodes of these modes for HSN_2_ (HSN_3_) are shown in Fig. 6(a–d) respectively. Obviously, it is clear that EELS maps can pick out each degenerate modes (*A*_1_ and *A*_2_, *C*_1_ and *C*_2_, *D*_1_ and *D*_2_). Based on the analysis of intrinsic charge distributions in Fig. 6(b,d), the features of coupling behaviors of these modes are same as HSN (*D*_3h_) case (discussed in above section). More details will be discussed later.

**Figure 7:**
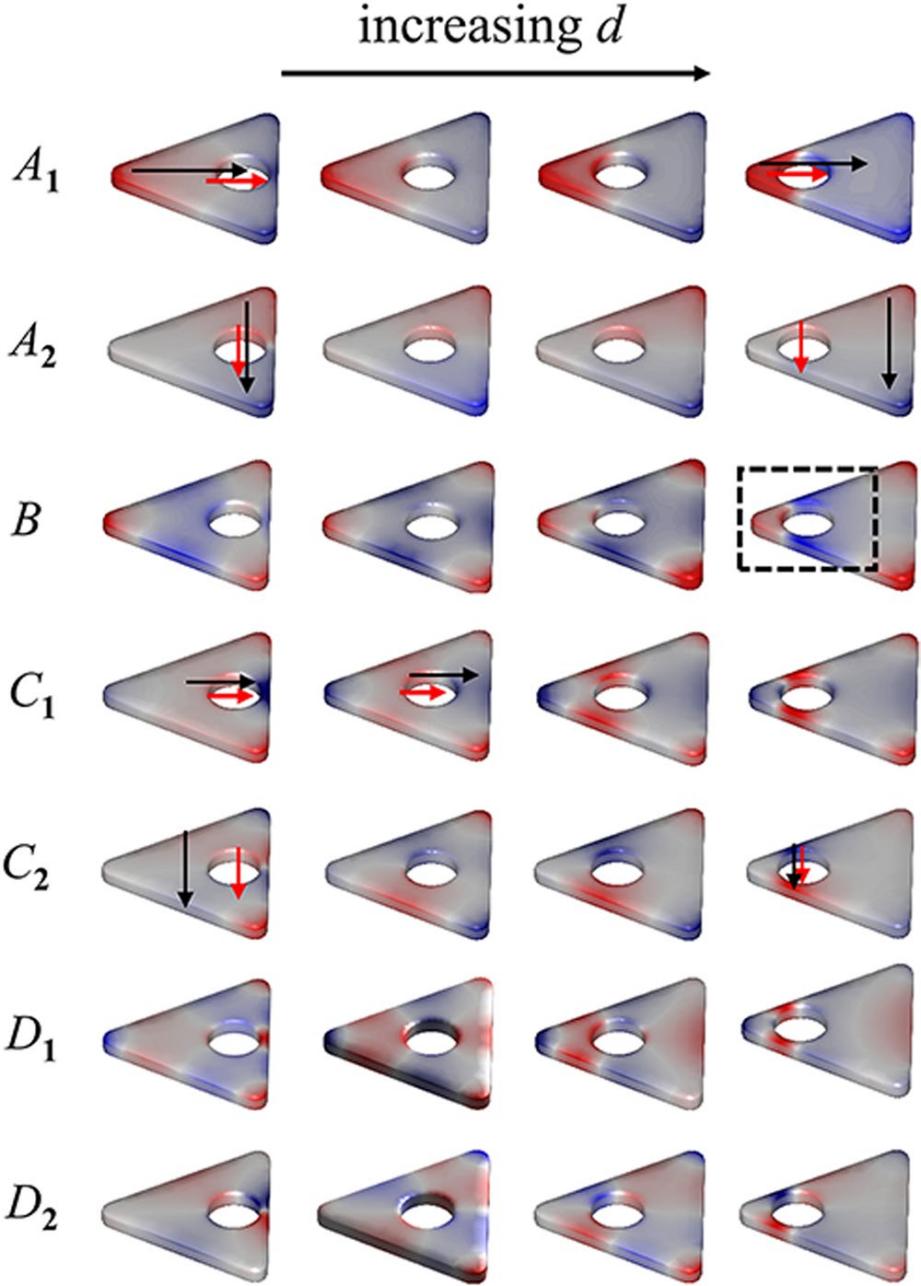
The eigenmodes (*A*_1_, *A*_2_, *B*, *C*_1_, *C*_2_, *D*_1_ and *D*_2_) of HSNs with different *d* (from left to right: 8, 16, 24, 32 nm).

To understand the effect of cavity position, EELS spectra of HSNs with different distances *d* (varying from 9 nm to 32 nm) are shown in Fig. 5(b,c). Here only EELS spectra taken at electron beam *b* and *c* cases are shown (EELS spectra for *a*-case have a few peaks and they can also be found in *b* or *c* cases). In Fig. 5(b,c), the white dashed line corresponds to HSN with *D*_3h_ symmetry (centers of inner and outer surface coincide). For other HSNs (*C*_2v_), it is clearly observed that the degenerate modes are separate: mode *A* splits into modes *A*_1_ and *A*_2_; the resonance energies of modes *B* and *C* become different (meanwhile, two degenerate states, *C*_1_ and *C*_2_, of mode *C* can also be distinguished); mode *D* splits into *D*_1_ and *D*_2_. Firstly, for modes *A*_1_ and *A*_2_, they show the opposite trend with increasing *d*: red-shift for mode *A*_1_ and blue-shift for mode *A*_2_. In the frame of dipolar-coupling model, the dipole-dipole (**p**_1_ and **p**_2_) interaction energy *W*_12_ is expressed by^53^

2 $${W}_{12}=\frac{{{\bf{p}}}_{1}\cdot {{\bf{p}}}_{2}-3({\bf{e}}\cdot {{\bf{p}}}_{1})({\bf{e}}\cdot {{\bf{p}}}_{2})}{{|{{\bf{r}}}_{1}-{{\bf{r}}}_{2}|}^{3}},$$

where **r**_1_ and **r**_2_ are the locations of **p**_1_ and **p**_2_ respectively, and **e** is the unit vector of Δ**r** = **r**_1_ − **r**_2_. With increasing *d*, |Δ**r**| will decrease for mode *A*_1_ and will increase for mode *A*_2_ (see *A*_1_ and *A*_2_ in Fig. 7). Thus, opposite trend of modes *A*_1_ and *A*_2_ can be understood. For mode *B*, there is no strong coupling until the inner surface approaching to the vertex. The strong interaction occurs when the cavity gets close to the vertex, and the coupling around vertex region is dipole-like interaction (mode *B* row, dashed box in Fig. 7). In Fig. 5(c), we also noticed that trend of mode *C*_1_ (*C*_2_) is similar to that of mode *A*_2_ (*A*_1_). As mentioned above, mode *C* has characteristic of dipole-dipole interaction ($\to \to$ for *C*_1_, $\uparrow \uparrow$ for *C*_2_). As *C*_1_ row shown in Fig. 7, |Δ**r**| will increase with *d*. This will result in weaker coupling (thus blue shift). Actually, quadrupole interaction occurs for larger *d* (i.e. rightmost one of *C*_1_ row). Conversely, the red-shifted behavior for mode *C*_2_ owing to |Δ**r**| increasing (see *C*_2_ row in Fig. 7). For degenerate mode *D* of HSN (*D*_3h_), it is derived from quadrupole-quadrupole coupling between inside and outside surfaces. For HSN (*C*_2v_) case, modes *D*_1_ and *D*_2_ are split due to the 3-fold rotational (*C*_3_) symmetry breaking (see *D*_1_ and *D*_2_ rows in Fig. 7). The interaction energy of quadrupole-quadrupole coupling is proportional to |Δ**r**|^−5^. Therefore, axisymmetric mode *D*_1_ is only excited by electron beam incidence in *b* or *a* case because of symmetrical distribution of applied electrical field along *α*-axis. And antisymmetric mode *D*_2_ is only excited by electron beam *c* case with asymmetrical distribution of applied electrical field along *α*-axis.

### Refractive index sensitivity of HSN

**Figure 8:**
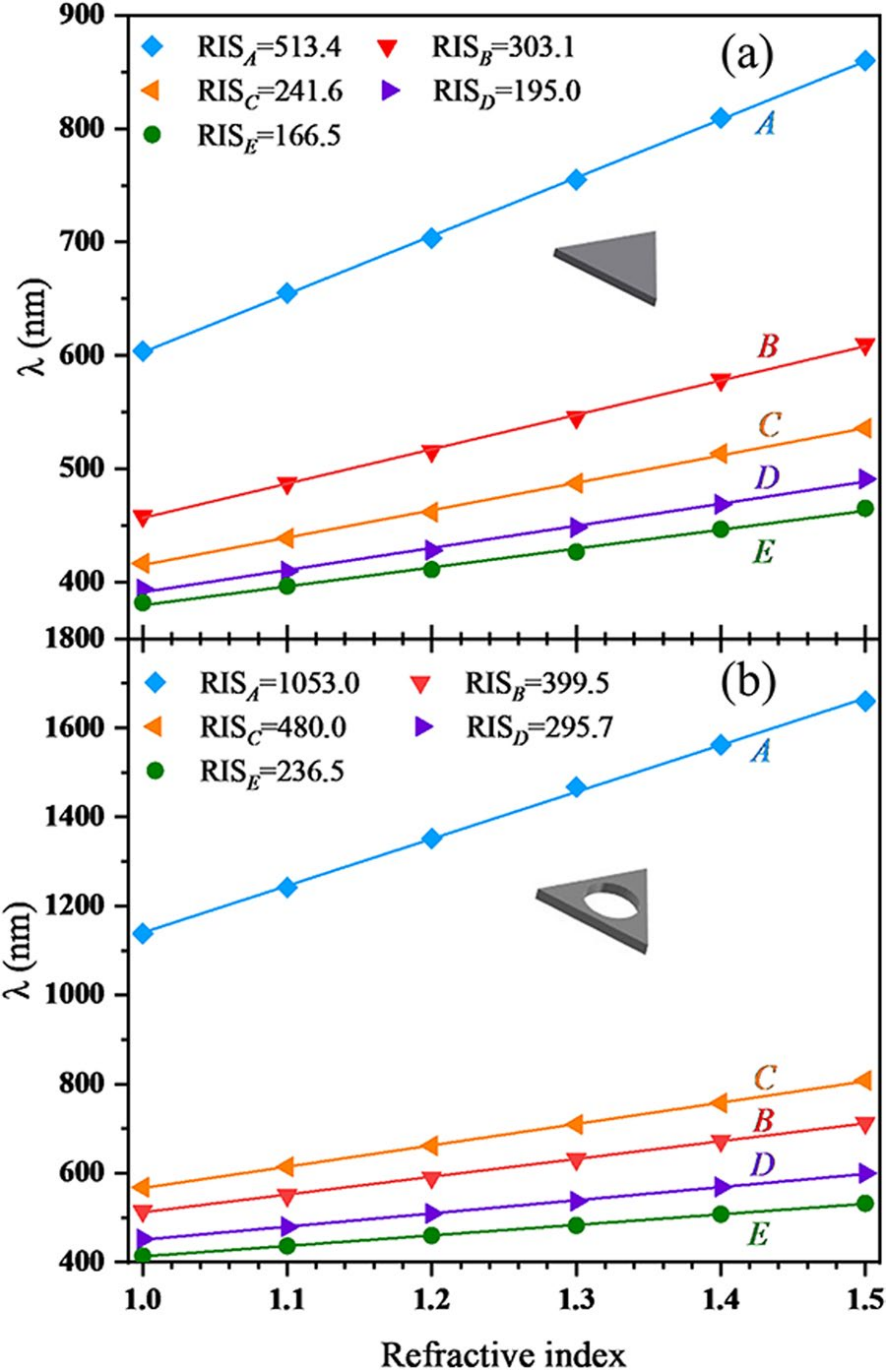
Relationship between the different LSP modes and the refractive index of surrounding medium for (**a**) SN case and (**b**) HSN_1_ case.

The resonance frequency of LSP mode also depends on the surrounding medium. In this section, we focus on the refractive index sensitivity (RIS) of HSN. Similarly, the influences of cavity size and position are discussed here. As mentioned above, RIS is defined as the shift in the wavelength (Δ*λ*) of LSP mode per unit in the refractive index sensitivity of the surrounding medium (RIS = Δ*λ/*Δ*n*). Figure 8 shows the shift in the plasmon wavelength as a function of the refractive index *n*. In SN case shown in Fig. 8(a), obviously, mode *A* has the highest RIS (513.4 nm RIU^−1^), and higher-order mode has the lower RIS. For HSN_1_ case (see Fig. 8(b)), the RIS of each mode can be significantly improved in comparison to SN case, especially for mode *A* (105.1% improvement) and *C* (98.7% improvement). Besides, in HSN_1_ case, the RIS of mode *C* (RIS_*C*_ = 480.0 nm RIU^−1^) is larger than that of mode *B* (RIS_*B*_ = 399.5 nm RIU^−1^). This is in opposite to SN case. Hazra^54^ *et al*. indicated that stronger coupling contributes to the greater RIS. It is clear that coupling strength of mode *C* is stronger than that of mode *B*, as discussed above. The influence of cavity size on RIS can refer to Table 1. Obviously, RIS for all these modes is increased with the cavity diameter. Previously, most works focused on the RIS of dipolar mode. The RIS values of single Ag nanosphere, nanocube and nanoprism have been reported to range from 160 to 240 nm RIU^−1^. Larger RIS value of 470 nm RIU^−1^ has been found for gold-coated silver nanoprisms^55^. EI-Sayed^43^ *et al*. reported the RIS of gold nanoframes around 620 nm RIU^−1^. To our knowledge, the highest RIS values reported are for nanobrach (1141 nm RIU^−1^) and nano bipyramids (1096 nm RIU^−1^)^39^. In contrast to the above works, the HSN (*D*_*3h*_) with large cavity size is a very promising candidate for sensing devices (i.e. 1053.0 nm RIU^−1^ for 2*r* = 30 nm). Meanwhile, high-order modes for HSN, such as mode *B* and *C*, also possess high RIS (303.1–399.5 nm RIU^−1^ for mode *B*, 241.6–480.0 nm RIU^−1^ for mode *C*).

**Table 1:** Dependence of the RIS for different modes on the cavity diameter 2*r* (HSN (*D*_3h_) case).

Cavity diameter 2*r* (nm)	RIS (nm RIU^−1^)				
	*A*	*B*	*C*	*D*	*E*
0	513.4	303.1	241.6	195.0	166.5
6	530.7	304.7	255.3	196.4	166.8
12	559.7	307.1	280.2	205.3	167.0
15	591.7	309.0	297.1	219.5	169.2
18	636.6	312.5	305.7	231.1	174.9
24	769.7	319.1	362.4	255.4	199.8
30	1053.0	399.5	480.0	295.7	236.5

**Figure 9:**
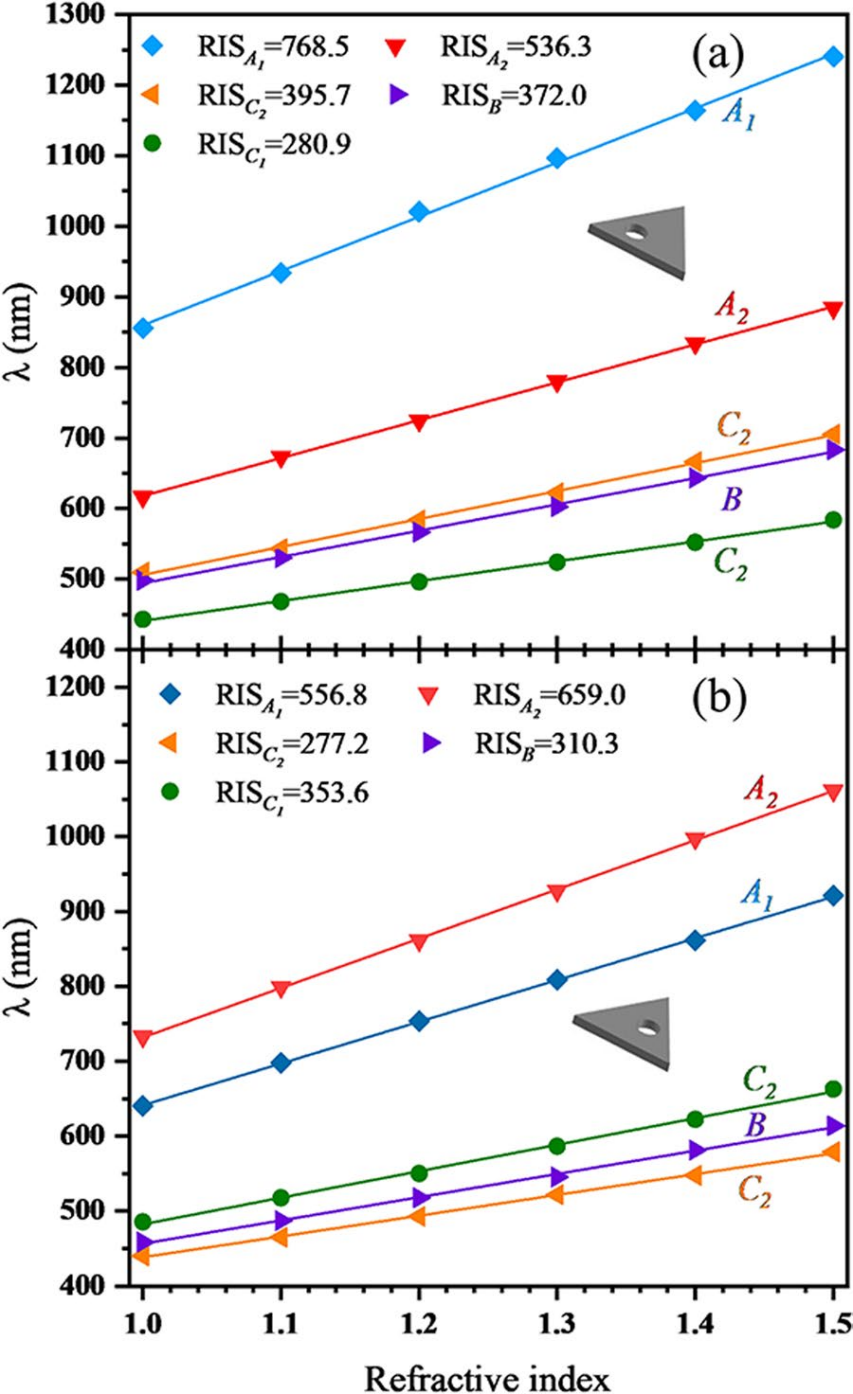
Relationship between the different LSP modes and the refractive index of surrounding medium for (**a**) HSN_3_ case and (**b**) HSN_2_ case.

Figure 9(a,b) show the RIS of the first five modes (*A*_1_, *A*_2_, *C*_2_, *B* and *C*_1_) for HSN_3_ and HSN_2_ respectively. The RIS of higher-order modes (*D*_1_, *D*_2_ and *E*) are lower and not shown here. In HSN (*C*_2v_) case, those degenerate modes are separate. The influences of cavity positions on the RIS of these modes also show differences. The RIS of mode *A*_1_ (${{\rm{RIS}}}_{{A}_{1}}=768.5$ nm RIU^−1^) is larger than mode *A*_2_ (${{\rm{RIS}}}_{{A}_{2}}=536.3$ nm RIU^−1^) for HSN_3_ case (see Fig. 9(a)). However, for HSN_2_ case, the phenomenon is opposite (${{\rm{RIS}}}_{{A}_{2}} > {{\rm{RIS}}}_{{A}_{1}}$). Actually, the RIS depends on the induced *E*-field intensity and distribution volume^56,57^. Higher *E*-field intensity and larger distribution range will make greater RIS. For HSN_3_ case, the coupling strength of mode *A*_1_ is stronger than mode *A*_2_, so is the *E*-field intensity. Thus ${{\rm{RIS}}}_{{A}_{1}}$ is larger than ${{\rm{RIS}}}_{{A}_{2}}$. On the other hand, the coupling strength of mode *A*_2_ will be stronger than mode *A*_1_ by decreasing *d*, and the RIS will be reversed. Similar behavior can be found for mode *C*_1_ and *C*_2_. The effect of cavity position on RIS can refer to Table 2. With increasing *d*, the alternation of coupling strength for mode *A*_1_ and *A*_2_ (*C*_1_ and *C*_2_) induces to opposite RIS trends. For large *d* (small *d*), RIS of mode *A*_1_ (*A*_2_) is larger than RIS of degenerate mode *A* (see 2*r* = 15 nm in Table 1.). In a sense, the RIS for HSN (*C*_2v_) will be improved compared with RIS for HSN (*D*_3h_). Therefore, changing the position of the cavity is also an effective method to improve RIS.

**Table 2:** Dependence of the RIS for different modes on the cavity position *d* (HSN (*C*_2v_) case)

Cavity position *d* (nm)	RIS (nm RIU^−1^)				
	*A*_1_	*A*_2_	*B*	*C*_1_	*C*_2_
9	556.8	659	310.3	353.6	277.2
12	571.6	614.4	310.3	310.3	283.1
18	602.5	586.2	311.1	291.9	305.3
24	649.3	563.1	311.5	269.5	335.5
30	707.4	538.3	350.6	270.9	372.8

**Figure 10:**
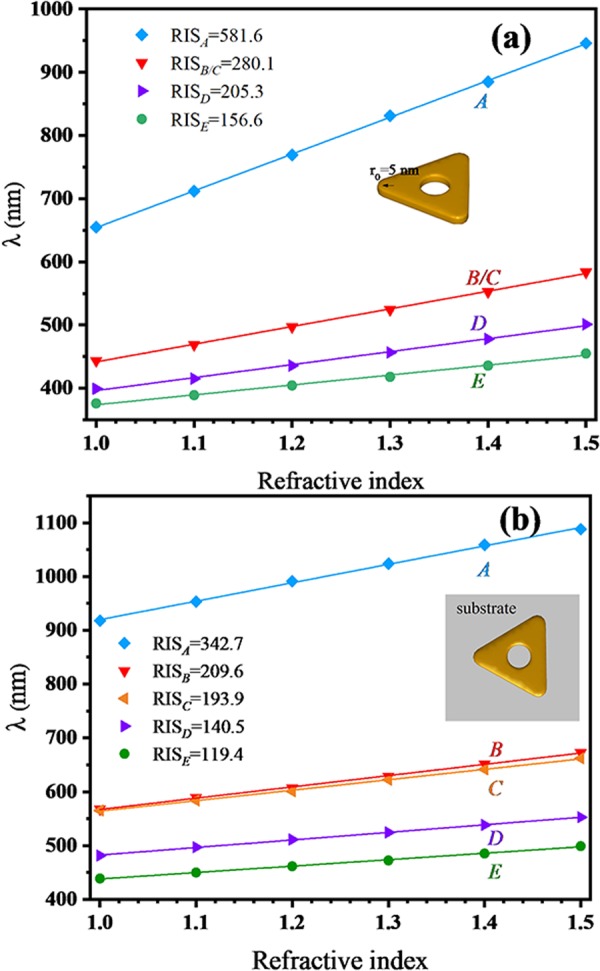
(**a**) Relationship between the different LSP modes and the refractive index of surrounding medium for HSN (2*r* = 15 nm, *r*_0_ = 5 nm); (**b**) relationship between the different LSP modes and the refractive index of surrounding medium for HSN (2*r* = 15 nm, *r*_0_ = 3 nm) placed on the substrate (*n* = 2).

In addition, the corner rounding effect is also a contributing factor for RIS. For all above HSNs, the circular arc radius of a corner *r*_0_ is set as 3 nm. Figure 10(a) shows the RIS of the first five modes (*A*, *B*, *C*, *D* and *E*) for a HSN with *D*_3h_ symmetry (2*r* = 15 nm, *r*_0_ = 5 nm). Here, when *r*_0_ = 5 nm, the energies of mode *B* and *C* are degenerate (mode B and C are very close for *r*_0_ = 3 nm case, see white dashed line in Fig. 2(b, c)). Obviously, in more rounded HSN (*r*_0_ = 5 nm) case, RIS values of these modes are lower than the previous one (*r*_0_ = 3 nm, refer to *2r* = 15 nm in Table 1). Thus, the corner rounding effect is unfavorable for high RIS, but it’s unavoidable during the chemically syntheses.

Furthermore, the effect of substrate on the RIS is also studied. Here the HSN (2*r* = 15 nm, *r*_0_ = 3 nm) is placed on a substrate whose refractive index *n* is set as 2. The RIS values of this HSN/substrate are shown in Fig. 10(b). Compared with the RIS of HSN without substrate (refer to *2r* = 15 nm in Table 1.), it is clear that the substrate effect is to lower the RIS of HSN. The decreased RIS is caused by the reduction of sensing area due to the existence of the substrate. Although the effects of rounding corner and substrate will lower RIS, it is an effective method to improve the RIS by tuning the size and position of the cavity of HSN.

## Conclusion

In summary, the LSP modes of hollow silver nanoprism have been studied by EELS simulation based on BEM. The effects of the cavity size and position are discussed. Firstly, EELS spectra of different HSNs (*D*_3h_) are calculated by varying cavity diameter (from 0 nm to 30 nm). Multipolar modes can be identified from EELS signals and each LSP mode is traced clearly. Assistant analyzes of eigenmodes show that mode *A*, *C* and *D* are degenerate. With increasing the cavity size, the red-shift phenomenon can be explained on the basic of the hybridization model. Besides, EELS spectra of different HSNs (*C*_2v_) with cavity diameter 2*r* = 15 nm are also performed by changing cavity position (*d* from 9 nm to 32 nm). In this case, the degenerate modes will be separate due to the misaligned centers of the cavity and the triangle (symmetry breaking). With changing the cavity position, the opposite trend of separate degenerate states are observed owing to different coupling nature. Furthermore, the refractive index sensitivities of HSNs, including the influences of the cavity size and position, are investigated. Increasing the cavity size and changing the cavity position can both improve the RIS. These results presented in our work show that HSNs are very promising candidates for the tunable plasmonic devices and sensors.

## Methods

The boundary element method (BEM) is a numerical method which has been used to solve electromagnetic field problems. In this case, arbitrary physical boundaries can be reconstructed by finite triangular or quadrilateral surface elements approximatively. In our work, we adopt the Matlab MNPBEM toolbox, which is developed by Hohenester and Trüler based on BEM^47,58^, to perform all of simulations. For nanoparticles, there is a close connection between LSP modes and eigenmodes of surface charge distributions. In the frame of BEM, the eigenmode equation can be given by:

3 $${{\rm{\oint }}}_{\partial {{\rm{\Omega }}}_{j}}{\rm{N}}\frac{\partial G({\bf{s}},{\bf{s}}{\boldsymbol{^{\prime} }})}{\partial n}\,{\sigma }_{i}({\bf{s}}{\boldsymbol{^{\prime} }})d{\bf{s}}{\boldsymbol{^{\prime} }}=2\pi {\lambda }_{i}{\sigma }_{i}({\bf{s}}),$$

the eigenenergy *λ*_*i*_ of the *i*th mode is derived from

4 $$\Re [{\rm{\Lambda }}({\omega }_{i})+{\lambda }_{i}]=0,$$

and

5 $${\rm{\Lambda }}(\omega )=2\pi \frac{{\varepsilon }_{2}(\omega )+{\varepsilon }_{1}(\omega )}{{\varepsilon }_{2}(\omega )-{\varepsilon }_{1}(\omega )},$$

where *ε*_1_(*ω*) and *ε*_2_(*ω*) are the dielectric functions of nanoparticle and surrounding environment, respectively. According to Eqs (3–5), it is obvious that the eigenmodes of nanoparticle only rely on its geometric shape; however, the eigenenergy is sensitive to the dielectric functions of nanoparticle and surrounding environment.

In STEM-EELS case, the electrical field of high-speed electrons act as external perturbation. For the trajectory of swift electron **r**(*t*) = **r**_0_ + **v***t*, with velocity ${\bf{v}}=v\hat{{\bf{z}}}$, the electric charge density can be written as:

6 $$\rho ({\bf{r}},\omega )=-\,\frac{e}{v}\delta ({\bf{R}}-{{\bf{R}}}_{0}){e}^{iq(z-{z}_{0})},$$

where **r** = (**R**, **z**), **R** denotes radial distance between point **r** and electron trajectory, **R**_0_ is the impact parameter in the *xy*-plane, and *q* = *ω*/*v*. The applied electric field generated by the fast electron can be expressed respectively as^59^:

7 $${{\bf{E}}}^{{\rm{app}}}({\bf{r}},\omega )=\frac{2e\omega }{{v}^{2}{\gamma }_{\varepsilon }\varepsilon }{e}^{i\omega z/v}[\frac{i}{{\gamma }_{\varepsilon }}{K}_{0}(\frac{\omega R}{v{\gamma }_{\varepsilon }})\hat{{\bf{z}}}-{K}_{1}(\frac{\omega R}{v{\gamma }_{\varepsilon }})\hat{{\bf{R}}}].$$

Here *K*_0_ is the modified 0-th order Bessel function, ${\gamma }_{\varepsilon }=1/\sqrt{1-\varepsilon {v}^{2}/{c}^{2}}$ is the Lorentz contraction factor. Notice that the energy of incident electron is fairly high (~100 keV), and much larger than the excitation energy of localized surface plasmon (a few eV energy). To calculate the energy loss of incident electrons, it is reasonable to assume that the incident direction of electrons is always along *z*-axis, and the speed remains unchanged. Thus, the energy loss of incident electrons arisen from the work against the induced field of nanoparticle can be given by^47,59^

8 $${\rm{\Delta }}E=e\int {\bf{v}}\cdot {{\bf{E}}}_{{\rm{ind}}}[{\bf{r}}(t),t]\,dt={\int }_{0}^{\infty }\hslash \omega \,{\Gamma }_{{\rm{EELS}}}({\bf{R}},\omega )\,d\omega ,$$

**E**_ind_ is the induced field, and Γ_EELS_ is the loss probability of the incident electrons. More numerical method details of Γ_EELS_ can be found in ref.^47^.

## Data Availability

The datasets generated and analysed during the current study are available from the corresponding author on reasonable request.

## References

[1] Ringe E, Sharma B, Henry A-I, Marks LD, Van Duyne RP (2013). Single nanoparticle plasmonics. Phys. Chem. Chem. Phys..

[2] Jain PK, Huang X, El-Sayed IH, El-Sayed MA (2007). Review of some interesting surface plasmon resonance-enhanced properties of noble metal nanoparticles and their applications to biosystems. Plasmonics.

[3] Amendola V, Pilot R, Frasconi M, Maragò OM, Iatì MA (2017). Surface plasmon resonance in gold nanoparticles: A review. J. Phys.: Condens. Matter.

[4] Maier, S. A. *Plasmonics: Fundamentals and Applications*. (Springer Science & Business Media, 2007).

[5] Maier SA, Atwater HA (2005). Plasmonics: Localization and guiding of electromagnetic energy in metal/dielectric structures. J. Appl. Phys..

[6] Cohen M, Shavit R, Zalevsky Z (2014). Observing optical plasmons on a single nanometer scale. Sci. Rep..

[7] Stiles PL, Dieringer JA, Shah NC, Van Duyne RP (2008). Surface-enhanced Raman spectroscopy. Ann. Rev. Anal. Chem..

[8] Kelly KL, Coronado E, Zhao LL, Schatz (2003). Schatz, G. C. The optical properties of metal nanoparticles: the influence of size, shape, and dielectric environment. J. Phys. Chem. B.

[9] Rosi NL, Mirkin CA (2005). Nanostructures in biodiagnostics. Chem. Rev..

[10] Hutter E, Fendler JH (2004). Exploitation of localized surface plasmon resonance. Adv. Mater..

[11] Akimov YA, Koh WS, Ostrikov K (2009). Enhancement of optical absorption in thin-film solar cells through the excitation of higher-order nanoparticle plasmon modes. Opt. Exp..

[12] Peng P (2017). Enhancing coherent light-matter interactions through microcavity-engineered plasmonic resonances. Phys. Rev. Lett..

[13] Xiao YF (2012). Strongly enhanced light-matter interaction in a hybrid photonic-plasmonic resonator. Phys. Rev. A.

[14] Kadkhodazadeh S (2014). Scaling of the surface plasmon resonance in gold and silver dimers probed by EELS. J. Phys. Chem. C.

[15] Scholl JA, García-Etxarri A, Koh AL, Dionne JA (2013). Observation of quantum tunneling between two plasmonic nanoparticles. Nano Lett..

[16] Schmidt FP, Ditlbacher H, Hofer F, Krenn JR, Hohenester U (2014). Morphing a plasmonic nanodisk into a nanotriangle. Nano Lett..

[17] Bigelow NW, Vaschillo A, Iberi V, Camden JP, Masiello DJ (2012). Characterization of the electron-and photon-driven plasmonic excitations of metal nanorods. ACS Nano.

[18] Barrow SJ, Rossouw D, Funston AM, Botton GA, Mulvaney P (2014). Mapping bright and dark modes in gold nanoparticle chains using electron energy loss spectroscopy. Nano Lett..

[19] Kociak M, Stéphan O (2014). Mapping plasmons at the nanometer scale in an electron microscope. Chem. Soc. Rev..

[20] Bosman M (2013). Surface plasmon damping quantified with an electron nanoprobe. Sci. Rep..

[21] de Abajo FG, Kociak M (2008). Probing the photonic local density of states with electron energy loss spectroscopy. Phys. Rev. Lett..

[22] Nicoletti O (2013). Three-dimensional imaging of localized surface plasmon resonances of metal nanoparticles. Nature.

[23] Hörl A, Trügler A, Hohenester U (2013). Tomography of particle plasmon fields from electron energy loss spectroscopy. Phys. Rev. Lett..

[24] Hörl A, Trügler A, Hohenester U (2015). Full three-dimensonal reconstruction of the dyadic Green tensor from electron energy loss spectroscopy of plasmonic nanoparticles. ACS Photon..

[25] Hörl A (2017). Tomographic imaging of the photonic environment of plasmonic nanoparticles. Nat. Commun..

[26] Rodríguez-Fernández J (2009). The effect of surface roughness on the plasmonic response of individual sub-micron gold spheres. Phys. Chem. Chem. Phys..

[27] Mazzucco S (2012). Ultralocal modification of surface plasmons properties in silver nanocubes. Nano Lett..

[28] Iberi V (2014). Resonance-Rayleigh scattering and electron energy-loss spectroscopy of silver nanocubes. J. Phys. Chem. C.

[29] Schaffer B, Hohenester U, Trügler A, Hofer F (2009). High-resolution surface plasmon imaging of gold nanoparticles by energy-filtered transmission electron microscopy. Phys. Rev. B.

[30] Sherry LJ, Jin R, Mirkin CA, Schatz GC, Van Duyne RP (2006). Localized surface plasmon resonance spectroscopy of single silver triangular nanoprisms. Nano Lett..

[31] Xu XB (2015). The influence of edge and corner evolution on plasmon properties and resonant edge effect in gold nanoplatelets. Phys. Chem. Chem. Phys..

[32] Cuche A (2015). Modal engineering of surface plasmons in apertured Au nanoprisms. Sci. Rep..

[33] Fromm DP, Sundaramurthy A, Schuck PJ, Kino G, Moerner W (2004). Gap-dependent optical coupling of single “bowtie” nanoantennas resonant in the visible. Nano Lett..

[34] Jain PK, Huang W, El-Sayed MA (2007). On the universal scaling behavior of the distance decay of plasmon coupling in metal nanoparticle pairs: a plasmon ruler equation. Nano Lett..

[35] Hooshmand N, Bordley JA, El-Sayed MA (2016). The sensitivity of the distance dependent plasmonic coupling between two nanocubes to their orientation: edge-to-edge versus face-to-face. J. Phys. Chem. C.

[36] Genç A (2016). Tuning the plasmonic response up: hollow cuboid metal nanostructures. ACS Photon..

[37] Yazdi S (2016). Reversible shape and plasmon tuning in hollow AgAu nanorods. Nano Lett..

[38] Mahmoud MA, Chamanzar M, Adibi A, El-Sayed MA (2012). Effect of the dielectric constant of the surrounding medium and the substrate on the surface plasmon resonance spectrum and sensitivity factors of highly symmetric systems: silver nanocubes. J. Am. Chem. Soc..

[39] Mock JJ, Smith DR, Schultz S (2003). Local refractive index dependence of plasmon resonance spectra from individual nanoparticles. Nano Lett..

[40] Stewart ME (2008). Nanostructured plasmonic sensors. Chem. Rev..

[41] Chen H, Kou X, Yang Z, Ni W, Wang J (2008). Shape-and size-dependent refractive index sensitivity of gold nanoparticles. Langmuir.

[42] Petryayeva E, Krull UJ (2011). Localized surface plasmon resonance: nanostructures, bioassays and biosensing—a review. Anal. Chim. Acta.

[43] Mahmoud MA, El-Sayed MA (2010). Gold nanoframes: Very high surface plasmon fields and excellent near-infrared sensors. J. Am. Chem. Soc..

[44] Zhi Y, Yu XC, Gong Q, Yang L, Xiao YF (2017). Single nanoparticle detection using optical microcavities. Adv. Mater..

[45] Johnson PB, Christy R-W (1972). Optical constants of the noble metals. Phys. Rev. B.

[46] Boudarham G, Kociak M (2012). Modal decompositions of the local electromagnetic density of states and spatially resolved electron energy loss probability in terms of geometric modes. Phys. Rev. B.

[47] Hohenester U (2014). Simulating electron energy loss spectroscopy with the MNPBEM toolbox. Comp. Phys. Commun..

[48] Nelayah J (2007). Mapping surface plasmons on a single metallic nanoparticle. Nature Phys..

[49] Nelayah J (2009). Direct imaging of surface plasmon resonances on single triangular silver nanoprisms at optical wavelength using low-loss EFTEM imaging. Opt. Lett..

[50] Losquin A (2015). Unveiling nanometer scale extinction and scattering phenomena through combined electron energy loss spectroscopy and cathodoluminescence measurements. Nano Lett..

[51] Keast VJ (2016). Higher order plasmonic modes excited in Ag triangular nanoplates by an electron beam. Plasmonics.

[52] Prodan E, Radloff C, Halas NJ, Nordlander P (2003). A hybridization model for the plasmon response of complex nanostructures. Science.

[53] Hohenester U, Krenn J (2005). Surface plasmon resonances of single and coupled metallic nanoparticles: A boundary integral method approach. Phys. Rev. B.

[54] Hazra B, Chandra M (2016). Plasmon hybridization mediated structure-specific refractive index sensitivity of hollow gold nanoprism in the vis-NIR negion. ACS Sensors.

[55] Aherne D, Charles DE, Brennan-Fournet ME, Kelly JM, Gun’ko YK (2009). Etching-resistant silver nanoprisms by epitaxial deposition of a protecting layer of gold at the edges. Langmuir.

[56] Haes AJ, Zou S, Schatz GC, Van Duyne RP (2004). Nanoscale optical biosensor: short range distance dependence of the localized surface plasmon resonance of noble metal nanoparticles. J. Phys. Chem. C.

[57] Jung LS, Campbell CT, Chinowsky TM, Mar MN, Yee SS (1998). Quantitative interpretation of the response of surface plasmon resonance sensors to adsorbed films. Langmuir.

[58] Hohenester U, Trügler A (2012). MNPBEM–A Matlab toolbox for the simulation of plasmonic nanoparticles. Comp. Phys. Commun..

[59] De Abajo FG (2010). Optical excitations in electron microscopy. Rev. Mod. Phys..

